# CRP and IL-6 as cardiovascular risk markers in diabetes

**DOI:** 10.6026/973206300221453

**Published:** 2026-03-31

**Authors:** Najmush Sahar Khan, Ravi Ramkishan Yadav, Shweta Kumari, Parth Jani, Jeremy Mathias Calderón Torres, Chennakesavulu Dara

**Affiliations:** 1 Department of Physiology, Santosh Deemed to be University, Ghaziabad, Delhi NCR, India; 2 Department of Biochemistry, TS Misra Medical College and Hospital, Amousi, Lucknow, Uttar Pradesh, India; 3 Department of Biotechnology, Savitribai Phule Pune University, Pune, Maharashtra, India; 4 Department of General Medicine, Government Medical College, Bhavnagar, Gujarat, India; 5 Department of Medicine, Pontifical Catholic University of Ecuador, Quito, Ecuador; 6 Department of General Medicine, ESIC Medical College, Hyderabad, India

**Keywords:** C-reactive protein (CRP), diabetes, interleukin-6 (IL-6), inflammation, cardiovascular risk

## Abstract

Cardiovascular diseases (CVD) are a leading cause of mortality in patients with diabetes mellitus and identifying biomarkers that predict cardiovascular risk is critical for early intervention. Therefore, it is of interest to explore the role of inflammatory markers, specifically CRP and IL-6, in predicting cardiovascular risk among diabetic patients. Elevated levels of both markers were found to be significantly associated with higher cardiovascular risk, as assessed by the Framingham Risk Score. Data show that CRP and IL-6 can serve as valuable biomarkers for identifying diabetic individuals at increased cardiovascular risk. This study advances knowledge by demonstrating that inflammation, as reflected by CRP and IL-6 levels, is an important predictor of cardiovascular events in diabetic patients.

## Background:

Cardiovascular diseases (CVD) are among the leading causes of morbidity and mortality worldwide, with diabetes mellitus being one of the primary risk factors for the development of CVD [1]. Diabetes, particularly type 2 diabetes, is associated with a significantly increased risk of cardiovascular events, including myocardial infarction, stroke and peripheral artery disease [2]. The mechanisms linking diabetes and cardiovascular risk are multifactorial, involving metabolic disturbances such as insulin resistance, hyperglycemia, dyslipidemia and inflammation. Among these, inflammation has emerged as a crucial player in the development and progression of cardiovascular complications in diabetic patients [3]. Inflammatory markers, such as C-reactive protein (CRP) and interleukin-6 (IL-6), have garnered significant attention in recent years due to their potential role in predicting cardiovascular risk. CRP is an acute-phase protein produced by the liver in response to inflammation and its levels are elevated in various inflammatory conditions, including diabetes [4]. IL-6 is a pro-inflammatory cytokine secreted by various cells, including adipocytes, endothelial cells and immune cells and it plays a key role in the inflammatory response associated with insulin resistance and atherosclerosis. Both CRP and IL-6 have been shown to be elevated in individuals with diabetes, particularly those with poor glycemic control and are thought to contribute to endothelial dysfunction, a key event in the development of atherosclerosis [5]. The link between inflammation and cardiovascular risk in diabetes is further supported by evidence showing that elevated levels of CRP and IL-6 are associated with the presence and progression of atherosclerosis. Atherosclerosis, the thickening and hardening of the arterial walls due to the accumulation of plaque, is a hallmark of cardiovascular disease and is closely linked to chronic inflammation [6]. In diabetic patients, hyperglycemia and insulin resistance lead to the activation of inflammatory pathways that contribute to endothelial injury, plaque formation and the rupture of atherosclerotic plaques, which can result in acute cardiovascular events [7]. Several studies have highlighted the predictive value of CRP and IL-6 in assessing cardiovascular risk in diabetic populations. For instance, elevated CRP levels have been found to be a strong predictor of future cardiovascular events in diabetic patients, even after adjusting for traditional risk factors such as blood pressure, cholesterol levels and smoking [8]. Similarly, IL-6 has been associated with an increased risk of cardiovascular mortality and morbidity in individuals with diabetes. These findings suggest that inflammatory markers may serve as useful biomarkers for identifying diabetic patients at higher risk for cardiovascular events, enabling more targeted interventions and better management strategies [9]. However, despite the growing body of evidence, the precise role of CRP and IL-6 in cardiovascular risk prediction in diabetes remains a subject of debate. While these markers are often elevated in diabetic patients, it is not yet fully understood whether their presence directly contributes to the development of CVD or if they simply serve as markers of underlying inflammation. Additionally, there is variability in the levels of these markers across different populations, which may limit their utility as universal predictors of cardiovascular risk [10]. Therefore, it is of interest to explore the role of inflammatory markers, specifically CRP and IL-6, in predicting cardiovascular risk in diabetic patients.

## Methodology:

This study aimed to evaluate the role of inflammatory markers (C-reactive protein [CRP] and interleukin-6 [IL-6]) in predicting cardiovascular risk in patients with diabetes mellitus. A cross-sectional design was employed and data were collected from a cohort of diabetic patients attending a tertiary care hospital. The study methodology is described below, including participant selection, data collection procedures, laboratory assessments and statistical analysis. The study involved 150 adult participants diagnosed with diabetes mellitus, aged between 30 and 70 years. The participants were selected from the outpatient and inpatient departments of a tertiary care hospital. A convenience sampling method was used to recruit eligible participants who met the inclusion criteria. The study included participants diagnosed with type 2 diabetes mellitus (T2DM) for at least one year, aged between 30 and 70 years and who consented to participate and provide blood samples. Individuals were excluded if they had a history of cardiovascular disease (CVD) prior to the study, current acute inflammatory conditions (e.g., infections, autoimmune diseases), severe renal or hepatic dysfunction, were pregnant or lactating, or had type 1 diabetes or other forms of diabetes. The sample size was calculated based on an estimated effect size of 0.3 for the correlation between inflammatory markers and cardiovascular risk, using G*Power software, which resulted in a minimum of 150 participants to achieve 80% power at a significance level of 0.05. Data were collected through structured questionnaires and patient medical records, capturing demographic (age, sex, BMI) and clinical characteristics (duration of diabetes, blood pressure, smoking history, family history of CVD). Glycemic control was assessed by measuring HbA1c levels, with a target range of <7.0% for optimal control. Inflammatory marker levels of CRP and IL-6 were measured using enzyme-linked immunosorbent assay (ELISA) techniques and participants were categorized based on their CRP and IL-6 levels. Cardiovascular risk was assessed using the Framingham Risk Score (FRS), which categorized participants into low, moderate and high-risk groups. Other laboratory tests included fasting blood glucose, lipid profile and serum creatinine levels. The study was approved by the institutional review board (IRB) and informed consent was obtained from all participants, ensuring confidentiality through anonymization of data and blood samples. Data analysis was performed using SPSS version 25, employing descriptive statistics, Pearson's correlation coefficient to assess relationships, multiple linear regression analysis to identify independent predictors, independent t-tests to compare CRP and IL-6 levels across risk categories and ROC curve analysis to determine the diagnostic value of CRP and IL-6. A p-value of <0.05 was considered statistically significant. Limitations include the cross-sectional design, which restricts the ability to establish causal relationships and reliance on a single-time point measurement of inflammatory markers, potentially missing long-term effects.

## Results:

A total of 150 participants diagnosed with type 2 diabetes mellitus (T2DM) were included in the study, with a mean age of 55.4 ± 8.7 years. Of these, 70% were male and 30% were female. The mean duration of diabetes was 8.3 ± 4.5 years. The analysis focused on evaluating the relationship between inflammatory markers (CRP and IL-6) and cardiovascular risk, as assessed by the Framingham Risk Score (FRS). Table 1 (see PDF) presents the demographic and clinical characteristics of the participants. Most of the participants had poor glycemic control, with a mean HbA1c of 8.2 ± 1.1%. The majority of participants had a history of hypertension (65%) and dyslipidemia (60%). The serum CRP and IL-6 levels were significantly higher in participants with high cardiovascular risk, as assessed by the Framingham Risk Score (FRS). The mean CRP level was 6.3 ± 2.4 mg/L and the mean IL-6 level was 9.5 ± 3.8 pg/mL. Elevated levels of CRP and IL-6 were positively correlated with higher FRS scores. There was a strong positive correlation between CRP levels and the Framingham Risk Score (r = 0.65, p < 0.001) and a moderate positive correlation between IL-6 levels and FRS (r = 0.53, p < 0.001). The correlation between both CRP and IL-6 with FRS was significant, indicating that higher levels of these inflammatory markers were associated with an increased risk of cardiovascular events. Multiple linear regression analysis was performed to assess the predictors of high cardiovascular risk in diabetic patients. Elevated CRP (β = 0.45, p < 0.001) and IL-6 (β = 0.35, p = 0.002) were found to be independent predictors of high cardiovascular risk, even after adjusting for other variables such as age, sex, HbA1c and lipid profile. Receiver Operating Characteristic (ROC) curve analysis was used to determine the diagnostic performance of CRP and IL-6 in predicting high cardiovascular risk. The area under the curve (AUC) for CRP was 0.84 (95% CI: 0.79-0.89) and for IL-6, it was 0.76 (95% CI: 0.71-0.81), indicating good diagnostic value. Table 2 (see PDF) shows the inflammatory marker levels in participants, with higher CRP and IL-6 levels observed in those at high cardiovascular risk compared to those at low and moderate risk. The correlation between these markers and the Framingham Risk Score (FRS) is detailed in Table 3 (see PDF), where CRP exhibits a strong positive correlation (r = 0.65, p < 0.001) and IL-6 a moderate positive correlation (r = 0.53, p < 0.001) with FRS. Table 4 (see PDF) highlights that both CRP and IL-6 are independent predictors of high cardiovascular risk in diabetic patients, with CRP showing the strongest association (β = 0.45, p < 0.001). Finally, Table 5 (see PDF) presents the results of the ROC curve analysis, which demonstrates that CRP (AUC = 0.84, 95% CI: 0.79-0.89) and IL-6 (AUC = 0.76, 95% CI: 0.71-0.81) are effective in predicting high cardiovascular risk, with CRP showing greater sensitivity and specificity.

## Discussion:

The results of this study indicate that elevated inflammatory markers C reactive protein (CRP) and interleukin 6 (IL 6) were significantly associated with increased cardiovascular risk in patients with type 2 diabetes mellitus (T2DM). This supports the hypothesis that chronic inflammation contributes to atherogenesis and cardiovascular complications in diabetes, consistent with evidence from previous research. Our findings generally align with prior studies examining inflammatory biomarkers and cardiovascular outcomes, though there are nuanced differences in methodology, populations and measured outcomes. Pradhan *et al.* (2017) [11] conducted a prospective cohort study in a high risk population (including diabetic individuals) and reported that elevated levels of CRP and IL 6 were significantly associated with a higher incidence of cardiovascular events such as myocardial infarction and stroke over a five year period. The study found that participants in the highest quartiles of CRP and IL 6 had notably greater hazard ratios for cardiovascular outcomes compared to those in the lowest quartiles, supporting the predictive role of these inflammatory markers for adverse cardiovascular events. Løfblad *et al.* (2021) [12] examined a population based cohort study examined inflammatory marker associations with cardiovascular outcomes and found that CRP was independently associated with cardiovascular mortality, with a graded increase in risk at higher CRP concentrations. In this study, the association persisted irrespective of diabetes status, highlighting that CRP remains a robust predictor of cardiovascular mortality across populations. Collectively, these studies corroborate our findings that CRP and IL 6 are important biomarkers in predicting cardiovascular risk. The mechanisms underlying these associations involve chronic low grade inflammation that contributes to endothelial dysfunction, plaque formation and progression of atherosclerosis-key processes in cardiovascular disease pathogenesis. Elevated IL 6 stimulates hepatic synthesis of acute phase proteins such as CRP, linking these biomarkers mechanistically [13].

## Conclusion:

We show the consistent evidence that elevated CRP and IL 6 are associated with increased cardiovascular risk, particularly in diabetic populations or high risk cohorts. Data show the utility of inflammatory biomarkers in cardiovascular risk stratification and highlight the importance of inflammation as a therapeutic target in diabetes management.
